# Management of Glycogen-Rich Clear Cell Carcinoma of the Breast: A Case Report

**DOI:** 10.7759/cureus.93429

**Published:** 2025-09-28

**Authors:** Joshua S Braganza, Baraah Mohamed, Cindy Presendieu, Nadj L Pierre, Heather Thieme

**Affiliations:** 1 Surgery, WellSpan York Hospital, York, USA; 2 General Surgery, Drexel University College of Medicine, Philadelphia, USA; 3 Surgical Oncology, WellSpan York Hospital, York, USA

**Keywords:** breast and endocrine surgery, breast cancer, breast cancer pathology, glycogen rich clear cell carcinoma, radiation & medical oncology

## Abstract

This case report presents an asymptomatic 75-year-old female with a screening mammogram revealing a left-sided breast lesion, ultimately diagnosed as glycogen-rich clear cell carcinoma (GRCC) following a comprehensive evaluation and histopathological confirmation. Initial ultrasound-guided biopsy suggested a papillary neoplasm, which was discordant, and was followed by a magnetic resonance imaging (MRI)-guided biopsy showing fibrocystic changes throughout and a complex sclerosing lesion with flat epithelial atypia. We proceeded with a lumpectomy without a sentinel lymph node biopsy. Final pathology revealed findings consistent with GRCC. A multidisciplinary discussion recommended forgoing axillary surgery and proceeding with radiation, hormonal therapy, and chemotherapy. This case highlights the challenges of diagnosing rare cancers, such as GRCC, and the importance of excisional biopsy in the diagnostic process. We also emphasize the importance of multidisciplinary tumor board discussions regarding rare lesions, such as GRCC, to help guide disease management and follow-up.

## Introduction

Invasive carcinoma with a glycogen-rich clear cell pattern (IC-GRCCP), formerly classified as glycogen-rich clear cell carcinoma (GRCC) of the breast, is a rare subtype of invasive breast carcinoma, accounting for approximately 0.9%-3% of all breast malignancies [1,2]. Invasive carcinoma with glycogen-rich clear cell cytoplasm of the breast was first described in 1981 [3]. It is defined as a neoplasm with an abundant amount of cytoplasmic glycogen granules that are periodic acid-Schiff (PAS) positive [4].

The etiology of GRCCP remains unclear, but it is believed by some to arise from ductal epithelial cells with altered glucose metabolism and dysfunction in the glycogen synthase pathway, leading to glycogen accumulation [5]. Risk factors are similar to those of invasive ductal carcinoma and include postmenopausal status, hormonal changes such as prolonged estrogen exposure, genetic mutations (e.g., BRCA1/2), and lifestyle factors, such as obesity.

Common clinical presentations of patients with IC-GRCCP include a palpable breast lump, changes in breast size or shape, nipple retraction, possible bloody discharge, and skin dimpling [2,6,7].

According to previous reports, multiple authors have documented estrogen receptor (ER)-negative, progesterone receptor (PR)-negative, and human epidermal growth factor receptor 2 (HER2)-positive cases, as well as triple-negative presentations, suggesting a heterogeneous nature [7]. In a review of several case reports and series, patients were typically referred for radiographic workup following the discovery of a palpable breast mass [1,2,6,7]. The current diagnostic approach aligns with that for most breast cancers, including diagnostic mammography, with or without ultrasound, and tissue biopsy as indicated. If mammography and biopsy findings are discordant, magnetic resonance imaging (MRI) and stereotactic biopsy may be considered [8].

Earlier literature noted a poor prognosis for IC-GRCCP; however, more recent studies suggest that outcomes depend on tumor grade, HER2 status, hormone receptor expression, and molecular characteristics [3]. The presence of intraductal components, solid papillary patterns, and genetic alterations may also contribute to the variability in clinical behavior and outcomes [1,9].

This case report aims to discuss the distinct histopathological and clinical features of GRCC and highlight the importance of multidisciplinary tumor board discussions in guiding the optimal management of this rare disease.

## Case presentation

A 75-year-old female presented to the surgical oncology clinic upon referral from her primary care provider after a routine screening mammogram detected a lesion in the left breast. Her prior oncologic history was notable for basal cell carcinoma of the right leg and left thigh, as well as melanoma of the right eyelid. All prior skin cancers had been treated surgically, and the patient was otherwise doing well. Her other medical conditions were non-contributory.

Her age at menarche was 13, and menopause occurred in her early 50s. She had her first child at 22 and did not breastfeed. There was no personal or family history of breast cancer. The only relevant family history of cancer was rectal cancer in her maternal grandmother at age 80. She had never undergone genetic testing. She was otherwise up to date with her mammograms and had one prior breast biopsy in 2002, which was benign.

**Figure 1:**
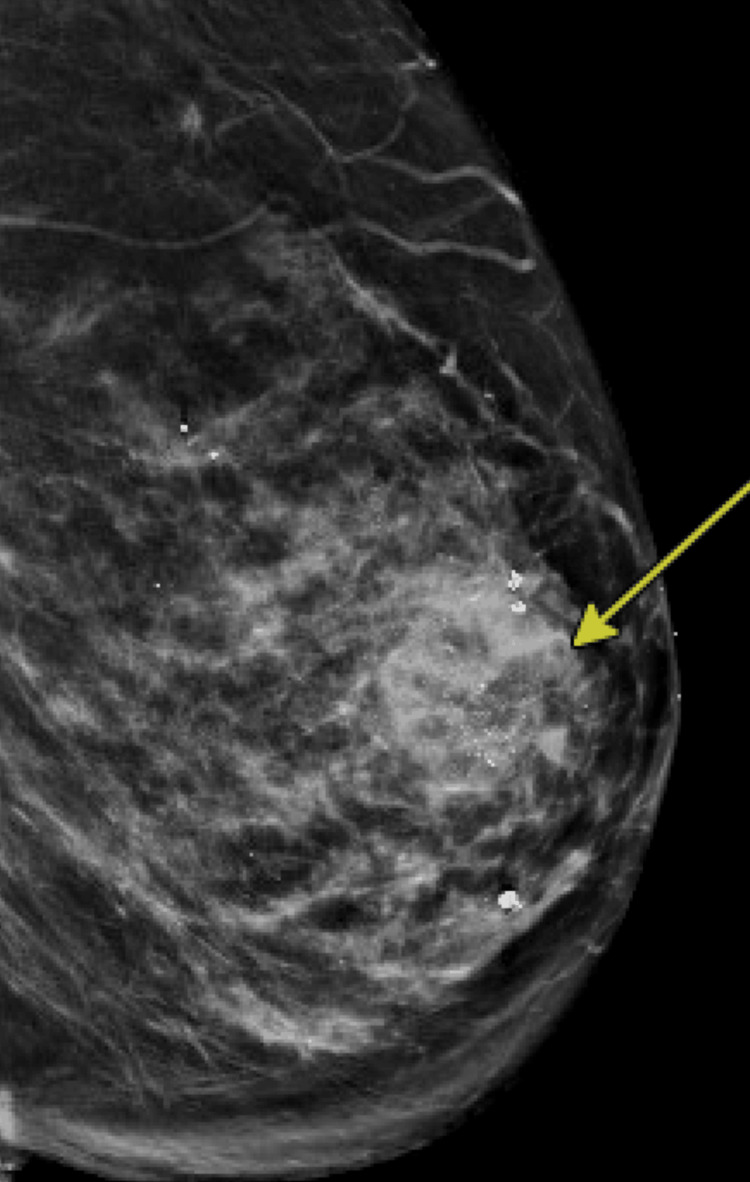
Diagnostic mammogram Arrow pointing to the BI-RADS 5 lesion BI-RADS 5, Breast Imaging Reporting and Data System 5

**Figure 2:**
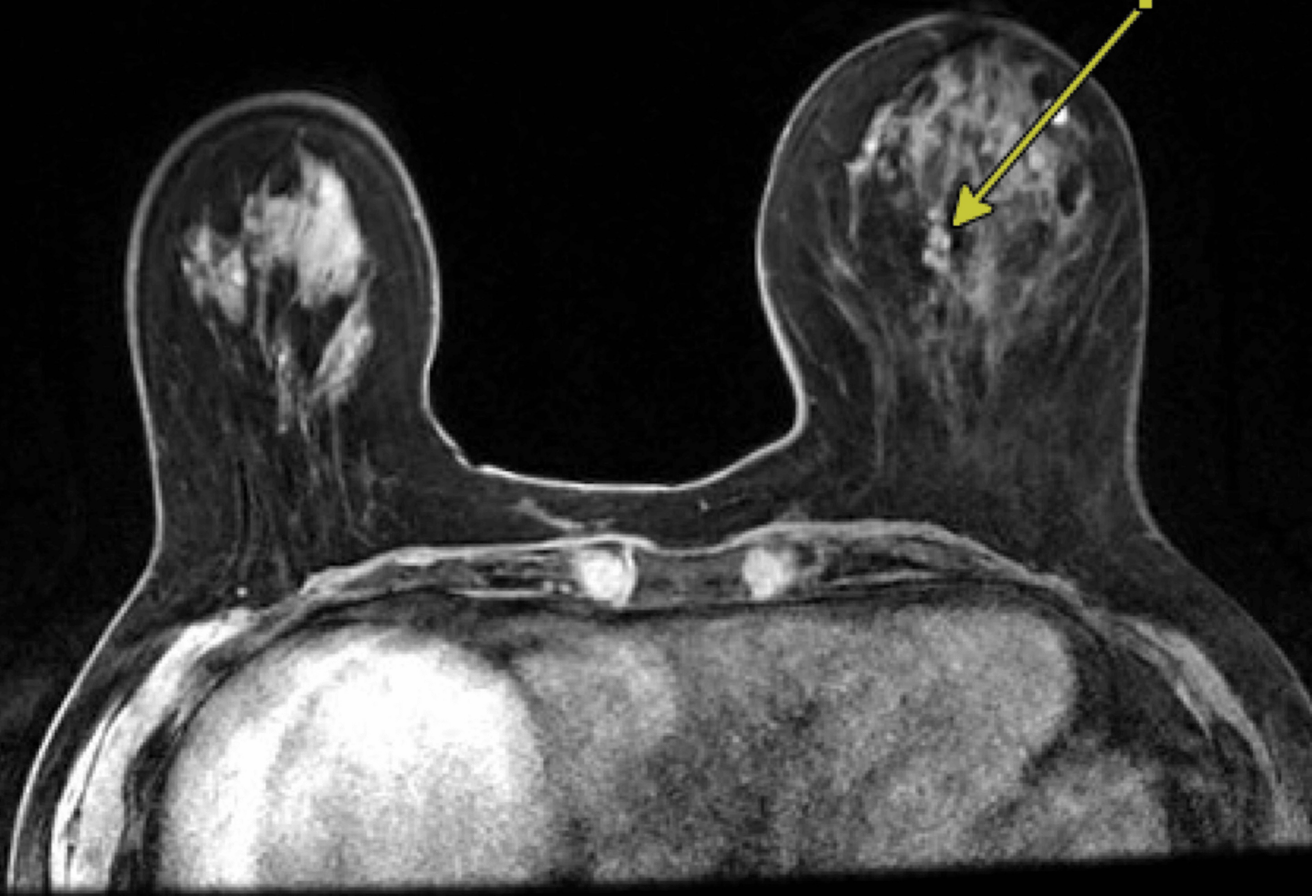
MRI redemonstrating a left-sided breast mass Arrow pointing to the mass for biopsy MRI, Magnetic Resonance Imaging

She underwent a diagnostic mammogram with ultrasound (Figure 1), which revealed a Breast Imaging Reporting and Data System 5 (BI-RADS 5) lesion, prompting an ultrasound-guided biopsy performed by our radiology team. Pathology from the biopsy showed an ER-positive, PR-negative papillary neoplasm, which was discordant with the BI-RADS 5 imaging findings. Given the discordance between the initial radiologic risk and the biopsy results, the patient subsequently underwent breast MRI (Figure 2), which revealed a 2 cm bilobed mass, concerning for possible ductal carcinoma in situ (DCIS). Based on these findings, she was referred to our surgical oncology clinic for further evaluation.

We recommended an MRI-guided biopsy, which was performed two weeks after her clinic visit. Pathology revealed fibrocystic changes throughout complex sclerosing lesions, radial sclerosing lesions, and flat epithelial atypia. The patient was informed of the results and consented to a lumpectomy without a sentinel lymph node biopsy, given the then-current diagnosis of a radial sclerosing lesion.

Final pathology revealed GRCC with ER positivity up to 91%-100%. PR expression was negative, with less than 10% of cells showing PR positivity. Her HER2 was initially equivocal; however, her fluorescence in situ hybridization found her specimen to be negative for HER2.

**Figure 3:**
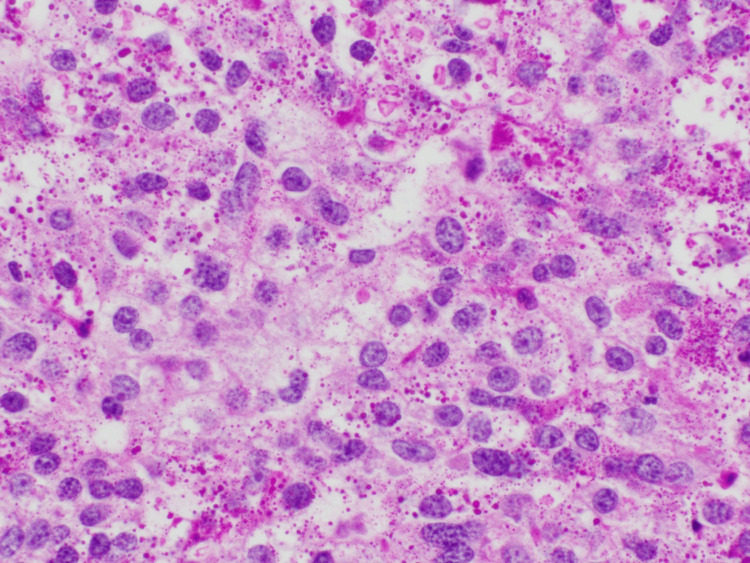
PAS staining highlighting the glycogen containing granules PAS, Periodic Acid-Schiff

Additionally, her Ki-67 score was 25%, and her Oncotype DX score was 52. In discussion with pathology, a second opinion was obtained to confirm the diagnosis. The PAS stain highlighted the glycogen granules seen in Figure 3. 

Given these results, her case was presented at the multidisciplinary tumor board for further recommendations. The decision was made to omit sentinel lymph node biopsy and initiate whole-breast radiation therapy, planned for 15 fractions. Chemotherapy was also recommended, consisting of docetaxel, cyclophosphamide, and granulocyte colony-stimulating factor (G-CSF) support every 21 days, followed by adjuvant letrozole.

Unfortunately, the patient completed only one cycle of chemotherapy due to an admission for neutropenic fever and declined further treatment. Notably, no source of infection was identified during hospitalization. She completed radiation therapy and continues on letrozole.

At her 10-month postoperative follow-up in the surgical oncology clinic, a repeat mammogram showed no concerning findings. She agreed to undergo a surveillance MRI in six months. She continues on aromatase inhibitor therapy and most recently followed up with her medical oncologist, who recommended initiation of ribociclib per the NATALEE trial, given her tumor’s T2 classification and grade 3 histology [10]. Ultimately, the patient declined. To date, she has had no breast-related complications and continues to tolerate letrozole well.

## Discussion

Historically, IC-GRCCP has been considered an aggressive subtype with a poor prognosis due to high nuclear grade, HER2 overexpression, and potential for lymph node metastasis. According to recent research, tumor pathology and hormone receptor status are important factors influencing patient outcomes. The patient in this case report was diagnosed with GRCC, characterized by ER positivity, PR negativity, and HER2 negativity. This is consistent with the limited receptor profile data available for this rare subtype of breast carcinoma [7].

Other cases have been reported with varying receptor profiles, including ER-negative, PR-negative, HER2-positive, and triple-negative phenotypes, among others. These combinations significantly affect prognosis; triple-negative tumors tend to have worse outcomes, whereas hormone receptor-positive tumors generally have more favorable prognoses. Further genetic studies of IC-GRCCP may improve our understanding of its biological behavior and aid in developing targeted treatments [1].

One of the initial challenges we faced in diagnosing GRCC was identifying radiographic features suggestive of this diagnosis. Our patient underwent three types of imaging - mammography, ultrasound, and MRI - due to discordant findings. Her mammogram revealed coarse heterogeneous calcifications, while ultrasound showed a dense mass. MRI demonstrated a bilobed enhancing mass.

According to the literature, there are no specific radiologic characteristics associated with GRCC. However, the most common mammographic finding is an irregular mass with microcalcifications. Ultrasound and MRI may reveal a microlobulated lesion with internal anechoic or cystic components [8].

Previous literature describes various histological patterns, including solid, papillary, micropapillary, and cribriform architectures. Some studies suggest that solid papillary patterns may be associated with more favorable outcomes [1,6,7]. In our case, the ultrasound-guided biopsy showed a papillary pattern with large cells containing clear cytoplasm, consistent with GRCC. The papillary pattern, along with ER positivity, may explain the favorable response observed in this patient. Additionally, Ki-67 remains a crucial prognostic factor in GRCC, as it can influence decisions regarding adjuvant therapy, as demonstrated in this case [3].

Current literature presents a controversial outlook on prognosis. Some case reports have shown favorable short-term outcomes in patients with GRCCP exhibiting a papillary pattern, particularly following simple mastectomy and sentinel lymph node biopsy [6]. Our patient underwent lumpectomy without sentinel lymph node biopsy, received 15 fractions of whole-breast radiation, started letrozole, and discontinued chemotherapy after one cycle due to neutropenic fever. Despite a more conservative surgical and medical approach, she remains healthy and shows no signs of recurrence at her one-year follow-up.

This variability in treatment approaches and outcomes highlights the importance of multidisciplinary tumor board discussions and tailoring therapy based on the patient’s unique histopathological features.

## Conclusions

GRCC is a rare disease with no uniform imaging characteristics, as noted in the current literature. Multiple imaging modalities may be needed, and the diagnosis needs to be confirmed by pathology. Microcalcifications in mammograms and lobulated lesions on ultrasounds and MRIs may hint at the diagnosis. Our case highlights the potential for more favorable outcomes with a solid papillary pattern, even when a conservative surgical approach is employed, such as lumpectomy without axillary lymph node dissection. This study emphasizes the importance of multidisciplinary discussion in the treatment of GRCC, given its variable hormone receptor profile and Ki-67 levels.
